# Unossified Membranous Strips of the Parietal Bone Mimicking a Fracture in a Child Presenting With Scalp Swelling: A Case Report

**DOI:** 10.7759/cureus.80034

**Published:** 2025-03-04

**Authors:** Dosti Regmi

**Affiliations:** 1 Pediatric Radiology, Le Bonheur Children's Hospital, Memphis, USA

**Keywords:** accessory suture, fracture, non-accidental trauma, parietal bone, unossified membranous bone

## Abstract

We report a case of a one-month-old male infant who was found to have a parietal scalp swelling on physical examination, with no reported history of trauma. Non-accidental trauma was suspected, and a skeletal survey was conducted to rule out any other traumatic injury. The skeletal survey showed a suspicious parietal fracture in the scalp swelling region. The rest of the survey was normal. CT scan was performed due to suspected non-accidental trauma. CT imaging demonstrated bilaterally symmetrical lucencies in the parietal bones extending perpendicular to the sagittal suture. Given its morphology and orientation, the lucency was determined to be a normal variant rather than a fracture. The subgaleal collection was not a recent hemorrhage and represented a late-appearing subdural effusion. This case highlights the importance of differentiating fractures from normal developmental variants of unossified strips of membranous bone, as misinterpretation can lead to significant medicolegal and emotional consequences.

## Introduction

Fractures are common in parietal bones in children and thus are the normal variants mimicking a fracture. The presence of any adjacent scalp swelling can make us more likely to call it a fracture [1,2]. The unossified strip of membranous bone in the parietal bone is a normal developmental variant due to incomplete mineralization in the parietal tissue, which is often bilateral and typically oriented perpendicular to sagittal suture and should not be mistaken for fractures [2]. Recognition of these variants is crucial in pediatric imaging to avoid misdiagnosis, especially in cases where non-accidental trauma is suspected.

## Case presentation

**Figure 1:**
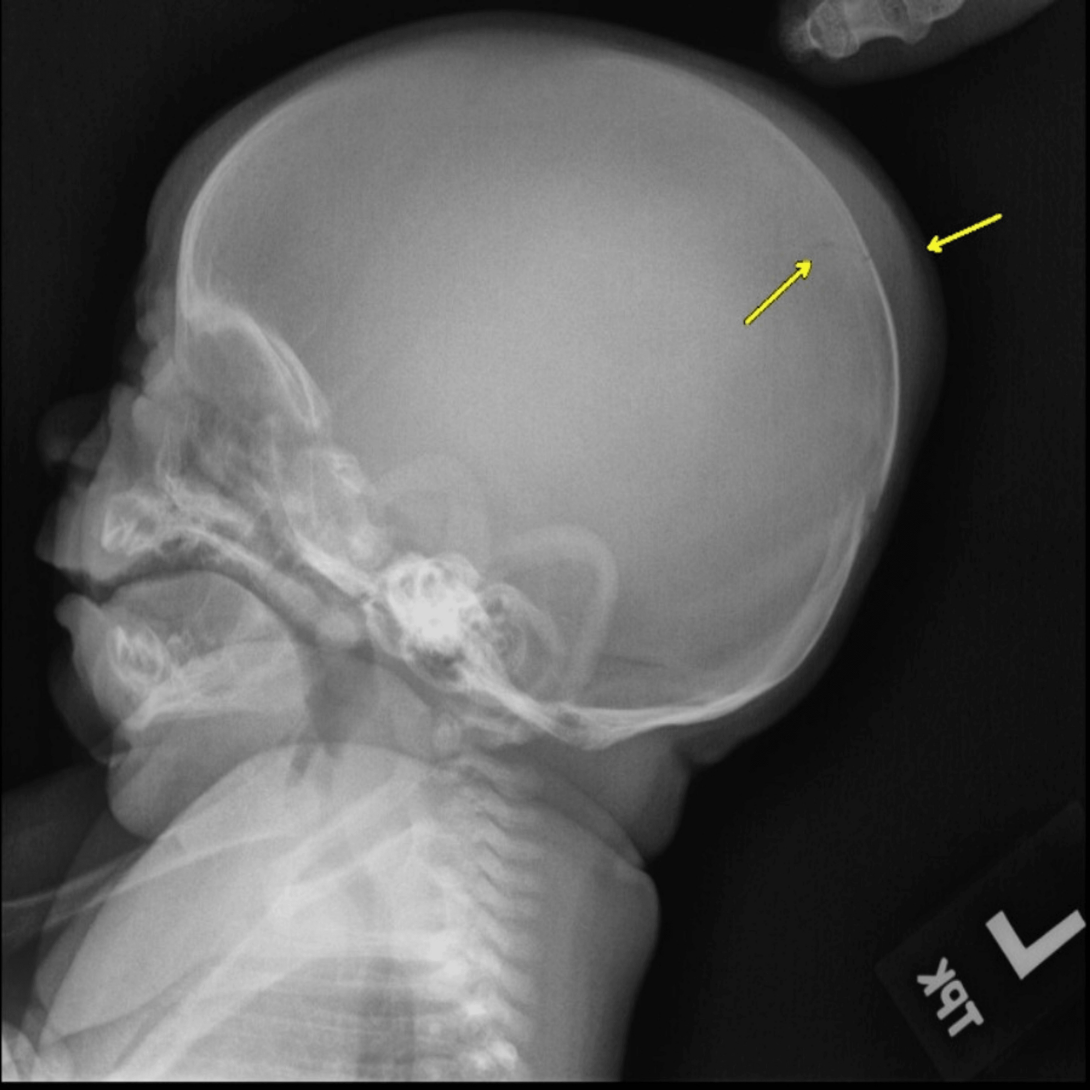
Lateral radiograph of the skull in the skeletal survey showing scalp swelling with underlying lucency in the parietal bone.

**Figure 2:**
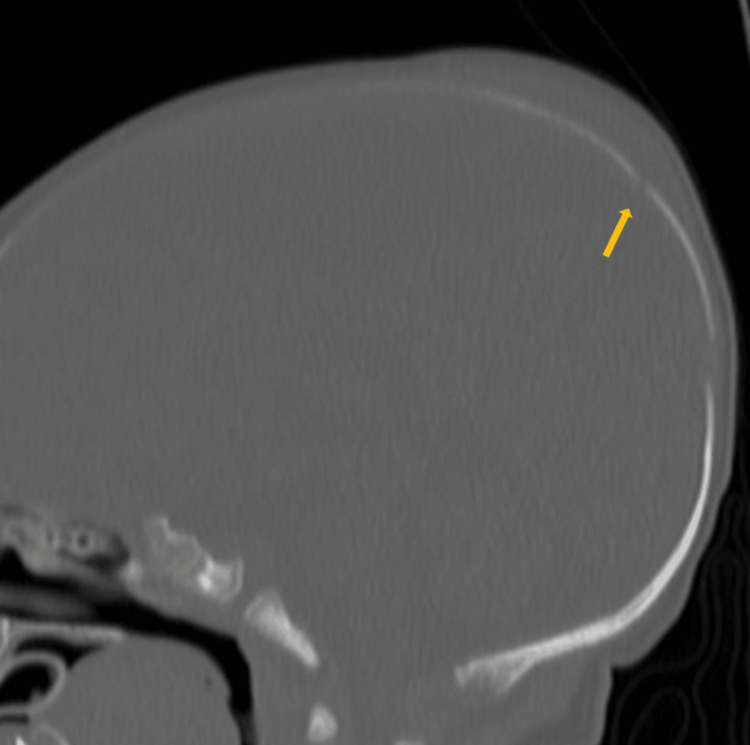
Sagittal CT bone window showing a lucency in the parietal bone with non-sclerotic margin and overlying scalp swelling.

**Figure 3:**
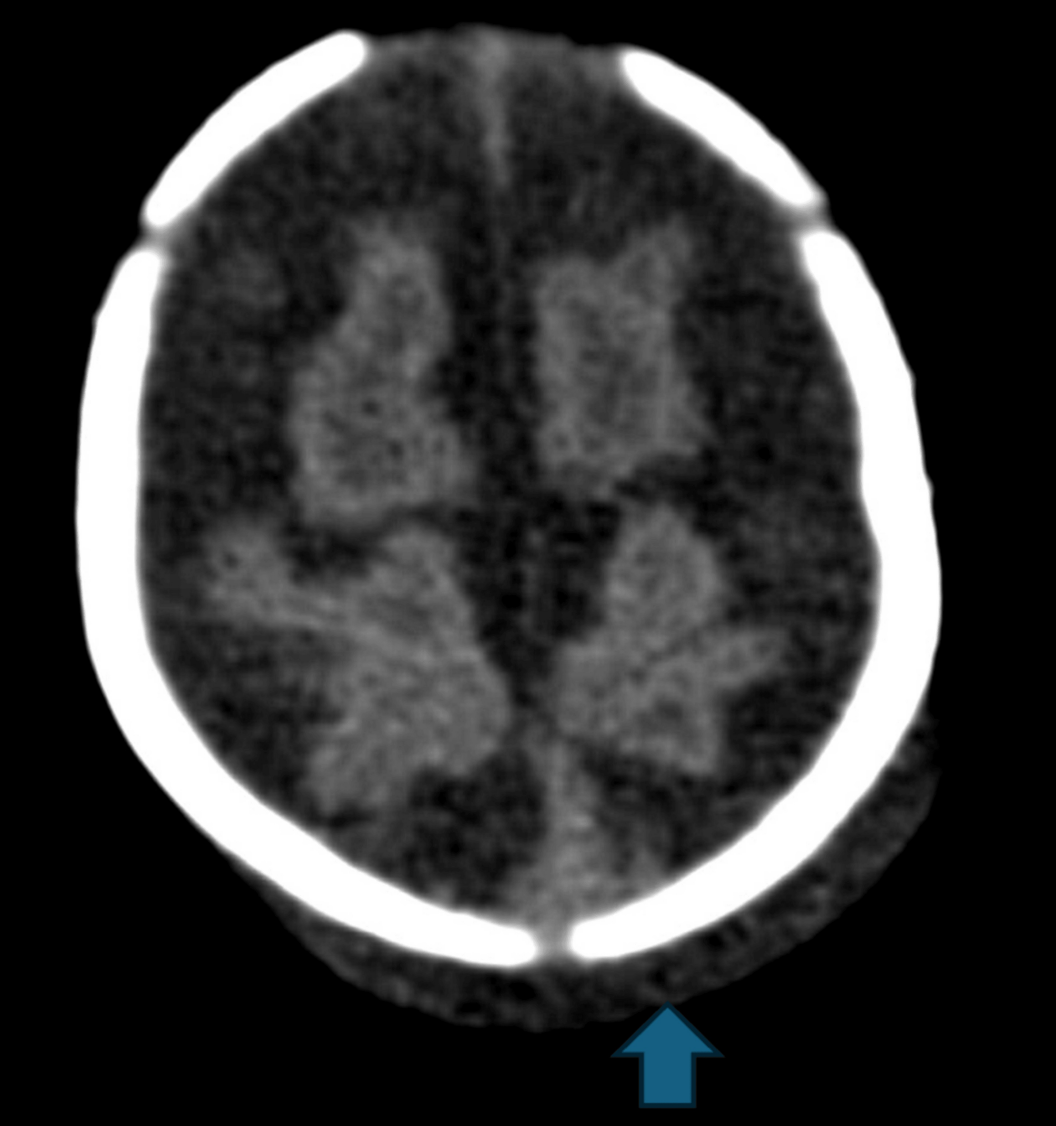
The axial head CT brain window showing parietal subgaleal collection with fluid attenuation crossing the suture line.

**Figure 4:**
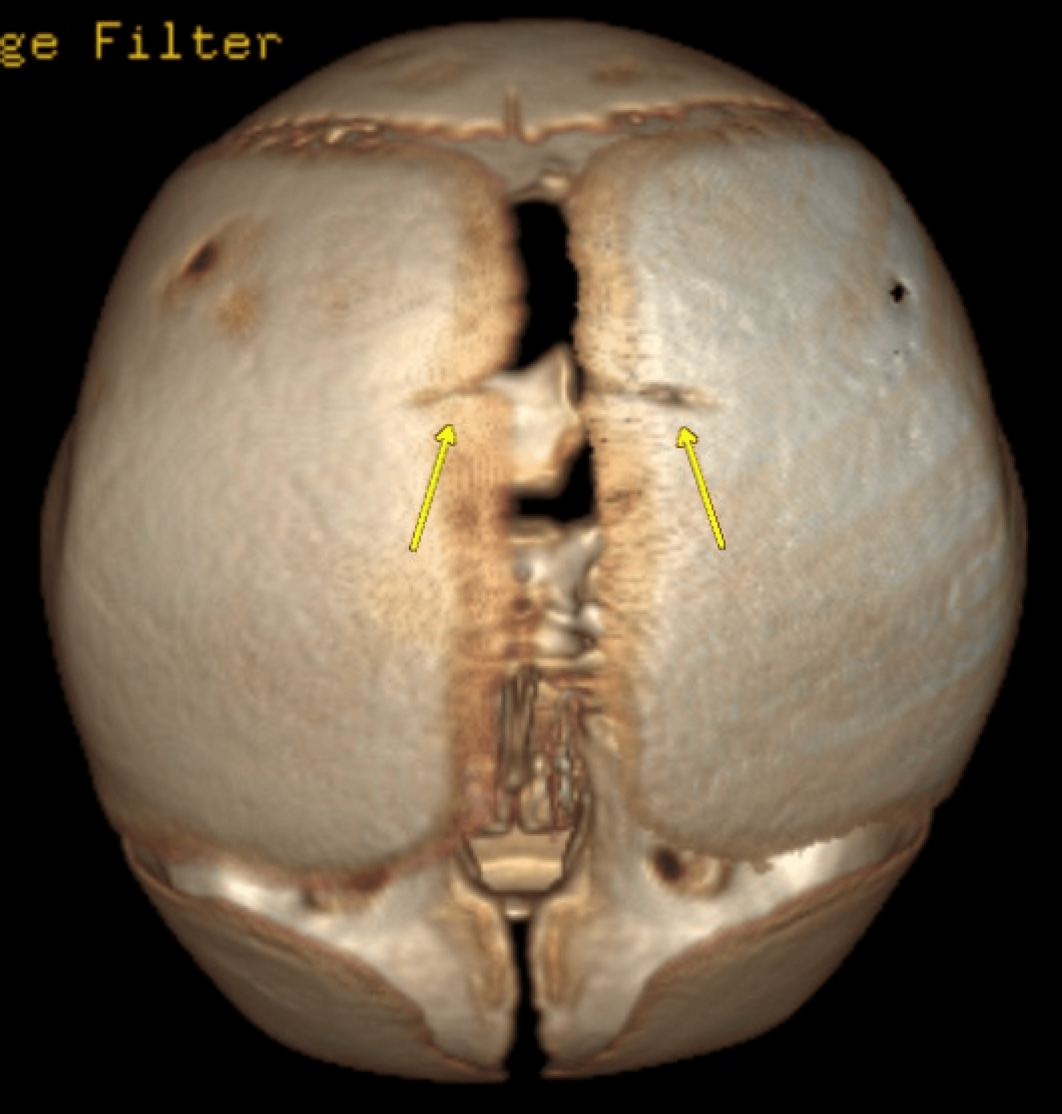
Three-dimensional reconstruction CT scan images of the skull showing bilaterally symmetrical lucencies in the parietal bones extending from the sagittal suture.

A one-month-old male child was found to have parietal scalp swelling during physical examination. The parents gave a history of prolonged vaginal delivery but did not require instrumentation. No known history of trauma was present. A skeletal survey was done for suspected non-accidental trauma. The lateral radiograph of the skull in the skeletal survey showed scalp swelling with underlying lucency in the parietal bone (Figure 1). This raised suspicion of skull fracture, and non-contrast CT of the head was ordered. The sagittal CT bone window showed lucent lines in the parietal bones with non-sclerotic margins and overlying scalp swelling was noted (Figure 2). The axial head CT brain window showed parietal subgaleal collection with fluid attenuation rather than acute hemorrhage (Figure 3). Three-dimensional reconstruction CT scan images of the skull showed bilaterally symmetrical lines in the parietal bones extending perpendicular to the sagittal suture (Figure 4). These lines represented unossified strips in the parietal bone, which is a variant anatomy encountered in the bones in the calvarium, which are formed by membranous ossification. The subgaleal collection was not acute hemorrhage but could represent unnoticed but resolving birth-related subdural effusion, which can normally persist for up to one to two months.

## Discussion

Parietal fractures are common in non-accidental trauma. Accessory sutures and aberrant ossification in this bone can mimic a fracture. Parietal bones can be formed from two centers, and the location of the accessory suture separating the bipartite parietal bone courses anterior to posterior, parallel to the sagittal suture, and is also called the sub-sagittal suture [2]. The lucency in our case was transversely oriented. It did not favor to be an accessory suture.

The bones in the cranial vault are formed from membranous ossification, and the sutures act as the major sites of bone growth and ossification along the leading margins of the cranial bones [3]. The linear strips of non-ossification perpendicular to the sutures can be seen in the developing membranous bones. A documented case of unknown cause of death had parietal bone lucency with no associated scalp or brain injury. The parietal bone was removed and fixated, and the histopathology showed unossified bone in that area, suggesting a strip of unossified membranous bone [1].

Fractures are often associated with scalp hematoma and underlying parenchymal contusion. The scalp swelling in our case could have misled us towards the diagnosis of the fracture. However, the CT showed that the collection was fluid-attenuating rather than hemorrhagic. Subgaleal effusion can present as soft, fluctuant scalp swelling manifesting after the first few weeks of life. Its exact etiology is not known, but it is noticed after a few weeks of birth. The late subgaleal effusion resolves spontaneously without any intervention [4].

Literature suggests that the fractures are well-defined rather than zigzag and do not have sclerotic margins. They usually get wider as they approach the suture lines, and in high-impact injuries, they can cross the suture line [2,5]. However, radiologists should have knowledge of the accessory sutures, synchondrosis, and variable appearance of developing membranous bones to misdiagnose them as fractures, which is sensitive medicolegally and can inflict a lot of unwarranted stress on the caregiver.

## Conclusions

This case underscores the need to differentiate normal anatomical variants from fractures in pediatric imaging. The parietal bone lucency was a developmental variant, not a fracture, highlighting the importance of recognizing unossified membranous strips in the bones of the calvarium. Accurate interpretation prevents misdiagnosis, unnecessary investigations, and caregiver distress.
